# Supplementary material for: HepatoPredict Accurately Selects Hepatocellular Carcinoma Patients for Liver Transplantation Regardless of Tumor Heterogeneity
Source: Cancers (Basel). 2025 Feb 2;17(3):500. doi: 10.3390/cancers17030500 (PMC11816190; doi:10.3390/cancers17030500)
Supplement: Supplementary file 1 [file cancers-17-00500-s001.zip › cancers-3412002-supplementary.pdf]

cancers

MDPI

Supplementary Material

Tables

Table S1. – Description of the currently used clinical criteria to select patients HCC for liver trans-plantation that were assessed in the study.

Criteria	Description
**Milan** [8]	1 tumor with diameter ≤ 5 cm or ≤ 3 tumors each with diameter ≤ 3 cm, and no macro-vascular invasion.
**UCSF** [36]	1 tumor with diameter ≤ 6.5 cm, or ≤ 3 nodules with the largest lesion with diameter ≤ 4.5 cm and total tumor diameter ≤ 8 cm.
**AFP Score** [37]	Largest tumor diameter: ≤ 3 cm [0 points]; 3 - 6 cm [1 point]; > 6 cm [4 points] + Number of tumors: 1 - 3 [0 points]; ≥ 4 [2 points] + AFP level: ≤ 100 ng/mL [0 points]; 100 – 1000 ng/mL [2 points]; > 1000 ng/mL [3 points]. Score ≤ 2.
**ArgScore** [38]	AFP > 100 ng/ml [Yes = 1 point, No = 0 point], tumor beyond Up-to-7 [Yes = 1 point, No = 0 point}. Score = 0 points [low risk].
**Warsaw** [39]	Expansion of Milan criteria including cases outside Milan criteria but within UCSF or Up to Seven (Up7) criteria with AFP < 100 ng/mL.
**MT2.0** [9]	Number of tumors and largest tumor diameter ≤ 7 + AFP < 200 ng/mL; Number of tumors and largest tumor diameter ≤ 5 cm + AFP < 400 ng/mL; Number of tumors and largest tumor diameter ≤ 4 cm + AFP < 1000 ng/mL.
**wALL** [40]	Intersection of the AFP Score and MT2.0.

Table S2. – Performance metrics of the retrained HepatoPredict algorithm comparing to its previous version[28]. The number of patients used to train each algorithm is indicated (n).

	Sensitivity (recall)	PPV (precision)	Specificity	NPV	Accuracy
**HepatoPredict previous version (n=162)**					
**HP Class I**	0.48	0.95	0.92	0.34	0.58
**HP Class II**	0.43	0.73	0.46	0.19	0.44
**HP Class I+II**	0.91	0.83	0.38	0.56	0.79
**HepatoPredict current version (n=232)**					
**HP Class I**	0.75	0.92	0.76	0.46	0.75
**HP Class II**	0.96	0.86	0.44	0.77	0.85

Table S3. – Performance metrics/accuracy measures of the retrained HepatoPredict algorithm and other currently used clinical criteria in the testing subsets.

	Sensitivity (recall)	PPV (precision)	Specificity	NPV	Accuracy	TP	FP	TN	FN
**ALL SAMPLES**									
**HP Class I**	0.75 ± 0.07	0.92 ± 0.04	0.76 ± 0.11	0.46 ± 0.09	0.75 ± 0.05	41 ± 5	4 ± 2	11 ± 3	14 ± 4
**HP Class II**	0.96 ± 0.03	0.86 ± 0.03	0.44 ± 0.13	0.77 ± 0.16	0.85 ± 0.04	52 ± 5	8 ± 2	7 ± 2	2 ± 1
**Milan** [8]	0.80 ± 0.04	0.82 ± 0.04	0.35 ± 0.09	0.33 ± 0.09	0.70 ± 0.04	44 ± 5	10 ± 2	5 ± 2	11 ± 3
**UCSF** [36]	0.91 ± 0.03	0.80 ± 0.04	0.16 ± 0.08	0.33 ± 0.16	0.75 ± 0.04	49 ± 5	13 ± 2	2 ± 1	5 ± 2
**AFP SAMPLES**									
**HP Class I**	0.76 ± 0.08	0.95 ± 0.04	0.81 ± 0.14	0.40 ± 0.12	0.77 ± 0.07	27 ± 4	1 ± 1	5 ± 2	9 ± 3
**HP Class II**	0.97 ± 0.03	0.91 ± 0.04	0.47 ± 0.19	0.74 ± 0.23	0.89 ± 0.04	34 ± 4	4 ± 2	3 ± 1	1 ± 1
**AFP Score** [37]	0.86 ± 0.04	0.87 ± 0.04	0.31 ± 0.16	0.29 ± 0.14	0.77 ± 0.04	31 ± 4	5 ± 2	2 ± 1	5 ± 2
**ArgScore** [38]	0.83 ± 0.05	0.88 ± 0.04	0.40 ± 0.18	0.31 ± 0.14	0.76 ± 0.05	29 ± 4	4 ± 2	3 ± 1	6 ± 2
**Warsaw** [39]	0.95 ± 0.03	0.86 ± 0.04	0.19 ± 0.14	0.42 ± 0.30	0.83 ± 0.05	34 ± 4	6 ± 2	1 ± 1	2 ± 1
**MT2.0** [9]	0.93 ± 0.04	0.86 ± 0.04	0.23 ± 0.15	0.37 ± 0.24	0.82 ± 0.05	33 ± 4	5 ± 2	2 ± 1	3 ± 1
**wALL** [40]	0.84 ± 0.05	0.87 ± 0.04	0.31 ± 0.16	0.27 ± 0.13	0.76 ± 0.05	30 ± 4	5 ± 2	2 ± 1	6 ± 2

Data represented as mean ± SD. The retrained HepatoPredict (HP) was compared with Milan criteria (Milan) and the University of San Francisco California (UCSF) criteria (n = 69), whereas the HP AFP samples subset was compared with AFP-based criteria such as AFP score, metroticket 2.0 (MT2.0), Argentinian Score (ArgScore), Warsaw criteria (Warsaw), and within all criteria (wALL) (n = 42). rHP Class I is a subset of rHP Class II. HP – retrained HepatoPredict, Milan – Milan criteria, UCSF – University of California San Francisco criteria, AFP – alpha-fetoprotein, ArgScore – Argentinian Score, Warsaw – Warsaw criteria, MT2.0 – metroticket 2.0 criteria, wALL – within all criteria, PPV – positive predictive value, NPV – negative predictive value, TP – true positives, FP – false positives, TN – true negatives, FN – false negatives.

Figures

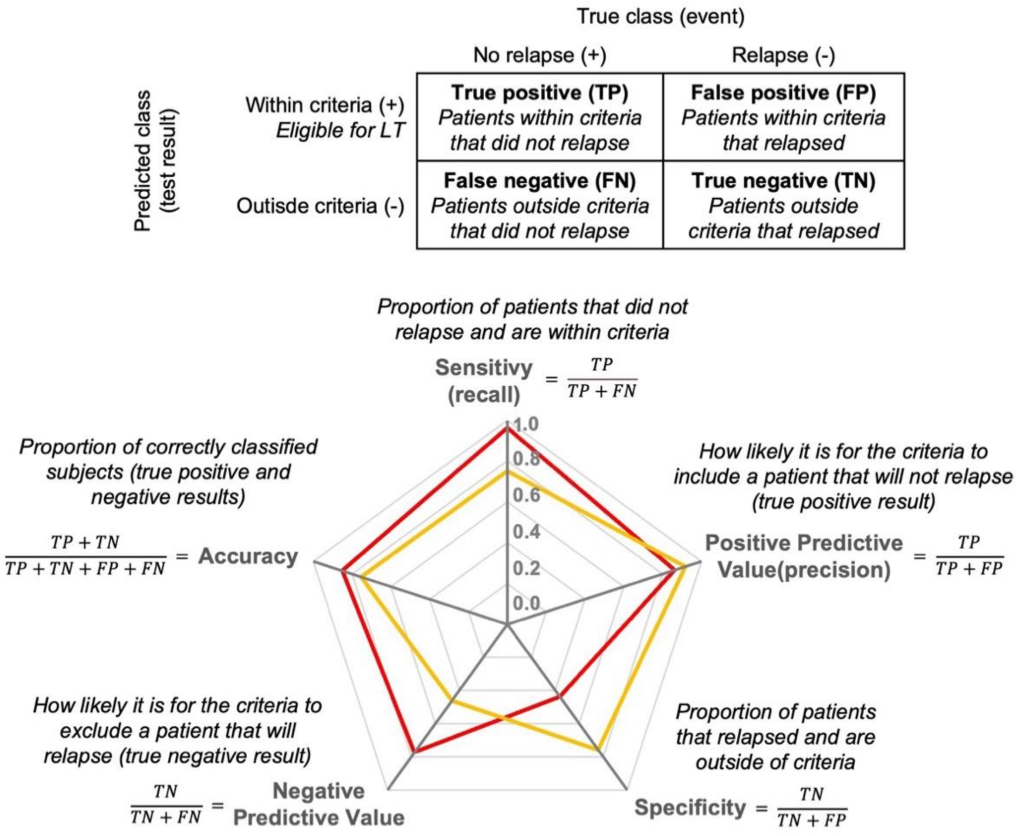

Figure S1. - Concepts and performance metrics employed in the study, including definitions and formulas.

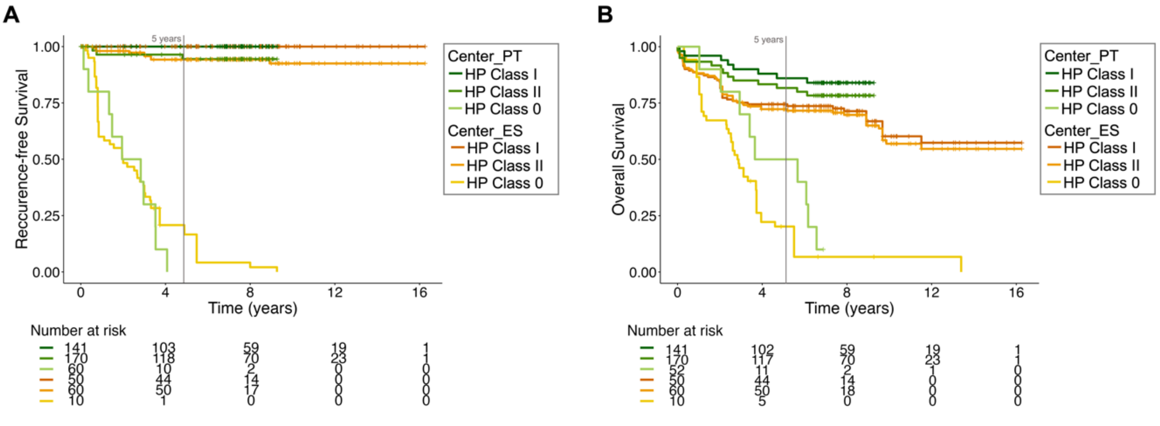

Figure S2. – Patients' recurrence-free survival and overall survival in the different cohorts. Comparison of the retrained HepatoPredict algorithm (HP) performance in patients from the Portuguese Center (Center_PT) – Hospital Curry Cabral, Lisbon, Portugal – and the Spanish Center (Center_ES) – Hospital Universitari i Politècnic La Fe, Valencia, Spain. Representation of the recurrence-free survival **(A)** and overall survival **(B)** curves and respective number of patients at risk at each timepoint. HP Class I is a subset of HP Class II. The log-rank test, based on OS analysis, showed no significant differences.

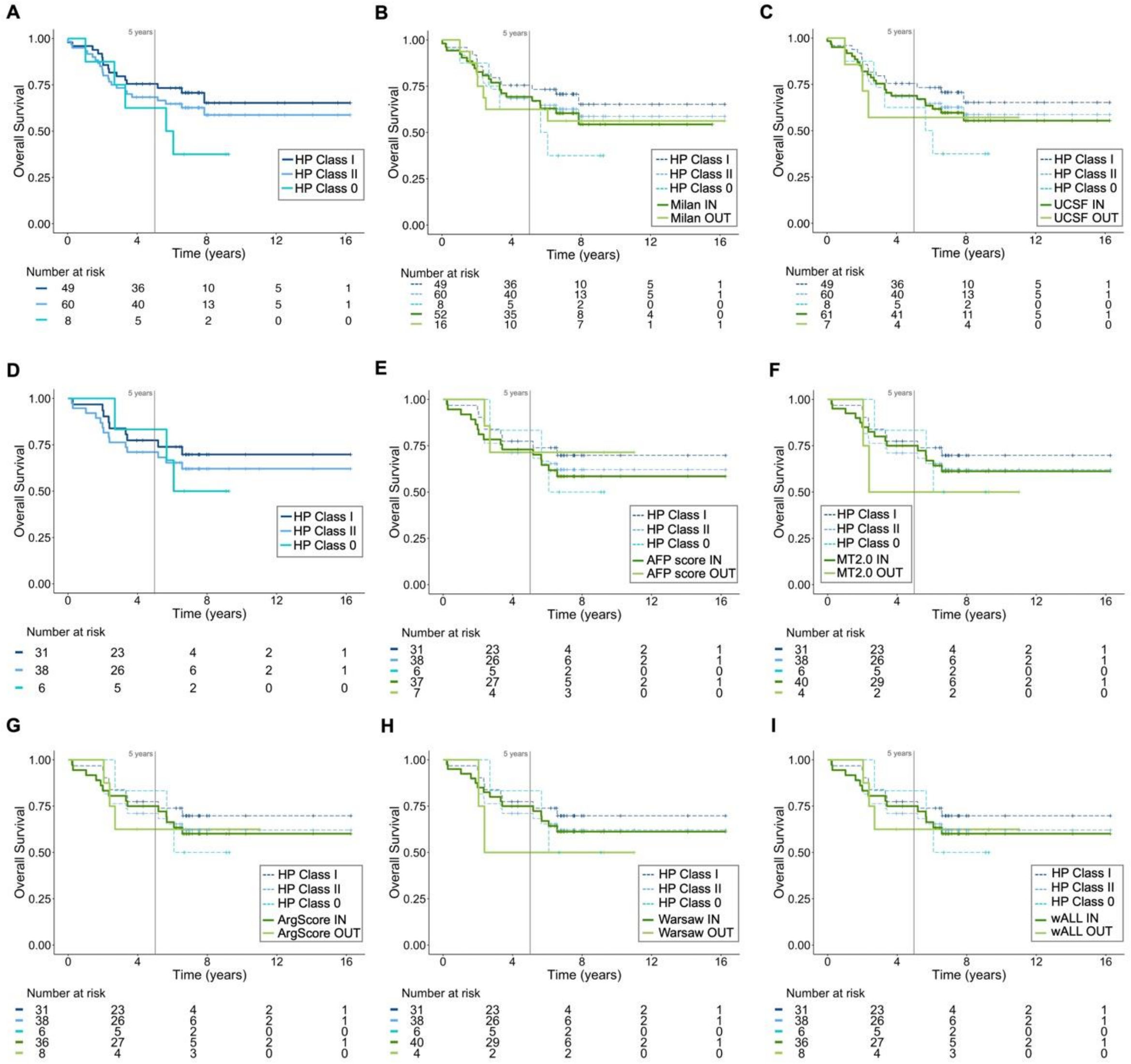

Figure S3. – Patients' overall survival according to different criteria. Overall survival curves (OS) of the most representative testing subset. The retrained HepatoPredict (HP) algorithm, Class I, II and 0 **(A)** was compared with Milan **(B)** and the University of San Francisco California (UCSF) **(C)** criteria, n = 68. OS was also calculated for the AFP samples subset of patients within the different HP classes **(D)** and compared with AFP-based criteria such as AFP score **(E)**, Metroticket 2.0 (MT2.0) **(F)**, Argentinian score (ArgScore) **(G)**, Warsaw **(H)**, and within all (wALL) criteria **(I)**, n = 41. For each criteria patients that are eligible (IN) and noneligible (OUT) for LT are represented. HP Class I is a subset of HP Class II. The log-rank test, based on OS analysis, showed no significant differences.

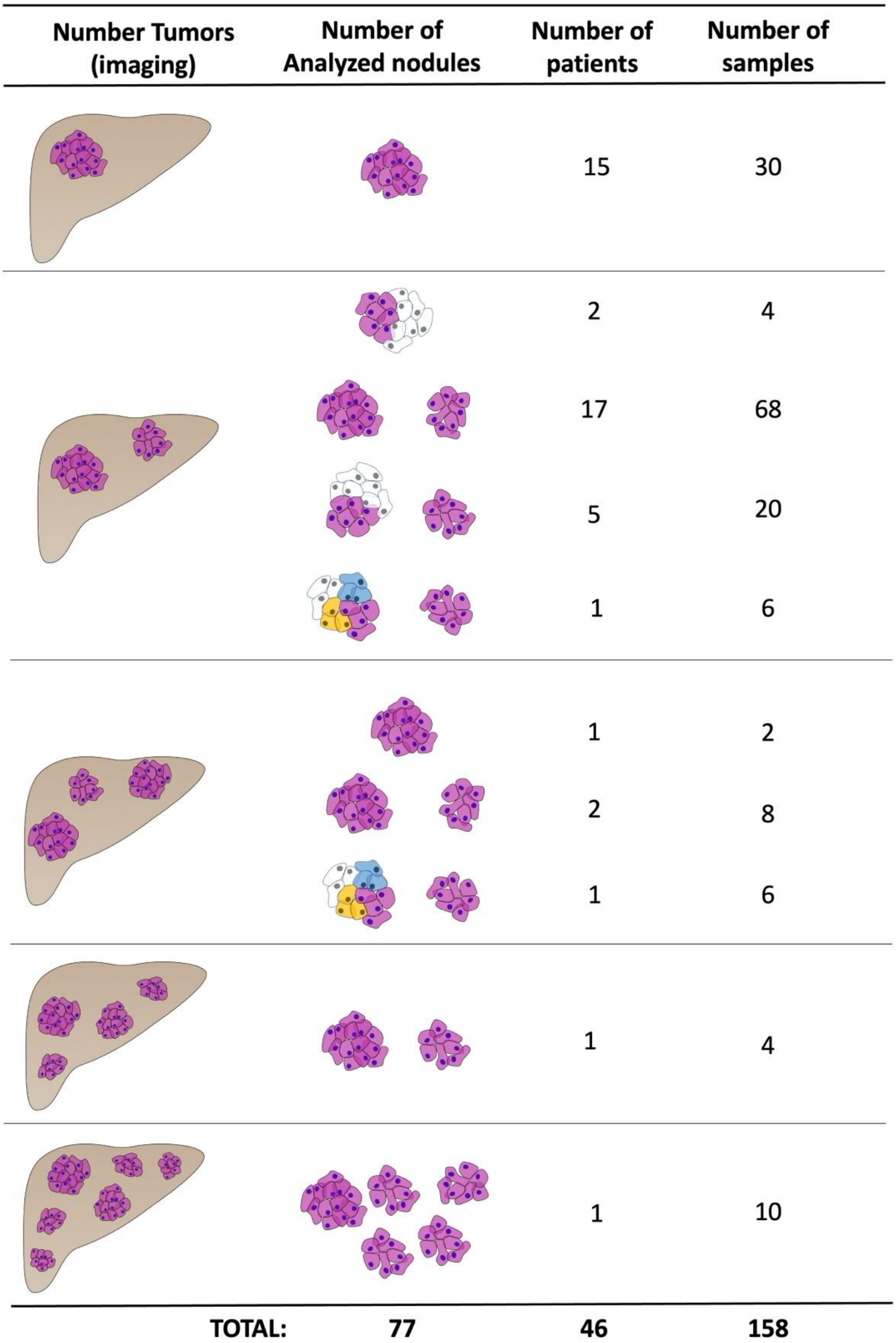

Figure S4. – Representation of dataset 2. Dataset 2 was composed of 46 patients diagnosed with HCC and submitted to LT, from which it was possible to isolate 158 independent tumor areas from 77 nodules. The first column – *Number of Tumors (imaging)* – represents the number of tumors identified pre-LT and used as a variable for the HP algorithm. The second column – *Analyzed nodules* – represents the nodules with available tissue. Different colors within each nodule illustrate tissue heterogeneity. The total number of nodules, patients and samples are depicted.

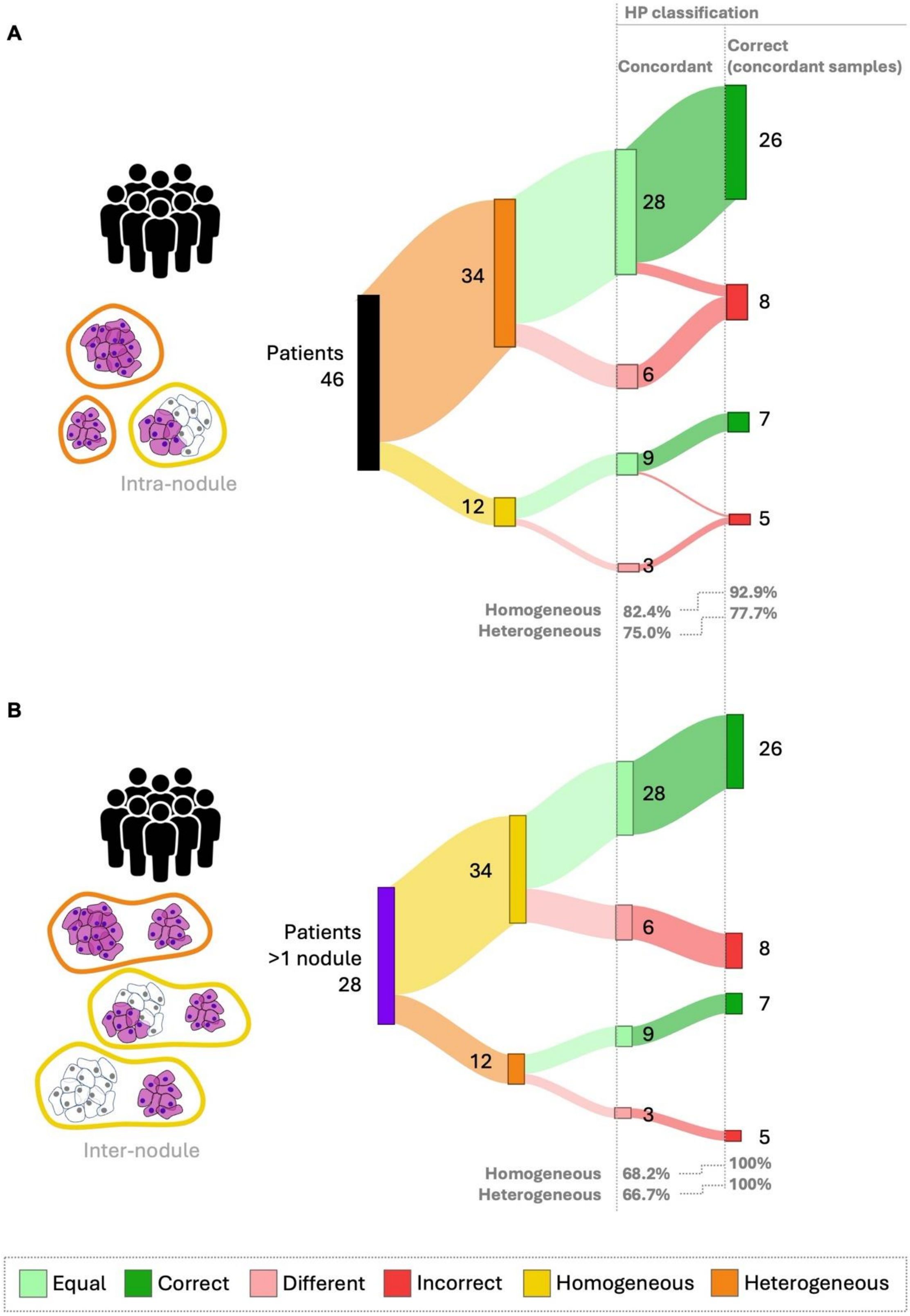

Figure S5. – HepatoPredict performance in the context of intra-nodule and inter-nodule heterogeneity – concordance and correct prediction in concordant samples. To evaluate the impact of intra-nodule and inter-nodule heterogeneity on HP performance, at least two samples of each nodule were collected and characterized regarding the concordance of their HP assay results specifically, whether these samples from the same nodule received the same HP classification. The performance of HP, defined as its ability to produce correct prognoses, was evaluated using only the HP concordant samples. The analysis was performed for homogeneous and heterogeneous patients, based on

intra-nodule (A) and inter-nodule (B) heterogeneity. The number (N) and percentage (%) are indicated for nodule/patients showing concordant vs. different HP results and for concordant samples. Any instance where the HP algorithm produced discordant results was automatically classified as incorrect.
